# Clinical and Genetic Characteristics of Congenital Hyperinsulinism in Norway: A Nationwide Cohort Study

**DOI:** 10.1210/clinem/dgae459

**Published:** 2024-07-04

**Authors:** Christoffer Drabløs Velde, Janne Molnes, Siren Berland, Pål Rasmus Njølstad, Anders Molven

**Affiliations:** Gade Laboratory for Pathology, Department of Clinical Medicine, University of Bergen, N-5020 Bergen, Norway; Mohn Center for Diabetes Precision Medicine, Department of Clinical Science, University of Bergen, N-5020 Bergen, Norway; Mohn Center for Diabetes Precision Medicine, Department of Clinical Science, University of Bergen, N-5020 Bergen, Norway; Department of Medical Genetics, Haukeland University Hospital, N-5021 Bergen, Norway; Department of Medical Genetics, Haukeland University Hospital, N-5021 Bergen, Norway; Mohn Center for Diabetes Precision Medicine, Department of Clinical Science, University of Bergen, N-5020 Bergen, Norway; Children and Youth Clinic, Haukeland University Hospital, N-5021 Bergen, Norway; Gade Laboratory for Pathology, Department of Clinical Medicine, University of Bergen, N-5020 Bergen, Norway; Mohn Center for Diabetes Precision Medicine, Department of Clinical Science, University of Bergen, N-5020 Bergen, Norway; Department of Pathology and Section for Cancer Genomics, Haukeland University Hospital, N-5021 Bergen, Norway

**Keywords:** hyperinsulinemic hypoglycemia, persistent hypoglycemia, genetic testing, monogenic disease, insulin dysregulation, neurologic sequela

## Abstract

**Purpose:**

Congenital hyperinsulinism (CHI) is a rare, monogenic disease characterized by excessive insulin secretion. We aimed to evaluate all probands with suspected CHI in Norway registered over the past 2 decades.

**Methods:**

The study included 98 probands. Clinical data were cumulated from medical records. All probands were screened for variants in the genes *ABCC8* and *KCNJ11*. Other CHI-related genes were Sanger-sequenced as indicated by the patients’ phenotype (n = 75) or analyzed by next-generation sequencing employing a panel of 30 CHI-related genes (n = 23).

**Results:**

Twenty-one probands (21%) received a diagnosis other than CHI, the most common being idiopathic ketotic hypoglycemia (9%) or syndromic hyperinsulinism (4%). In the final cohort of 77 CHI probands, genetic findings were revealed in 46 (60%). *ABCC8* variants were most common (n= 40), and 5 novel variants were identified. One proband harbored both the pathogenic *GCK* variant p.(Ala456Val) and the *ABCC8* variant p.(Gly505Cys). Although most *ABCC8* variants caused immediate disease onset with severe hypoglycemia and were diazoxide-unresponsive, 8 probands had a heterozygous, apparently dominant variant with milder phenotype. Two probands had pathogenic variants in *GLUD1*, whereas variants in *HADH*, *HNF4A*, *KCNJ11,* and *HK1* were identified in 1 proband each, the latter being noncoding. Neurologic sequelae were reported in 53% of the CHI probands. Of nonsurgically treated probands, 43% had spontaneous resolution. The minimum birth prevalence of CHI in Norway is 1:19,400 live births.

**Main Conclusion:**

Individuals with disease-causing *ABCC8* variants dominated our cohort. Patients with known genetic etiology had earlier and more severe disease onset than genetically unsolved patients.

Congenital hyperinsulinism of infancy (CHI) is a rare, monogenic disease where the hallmark is inappropriately increased insulin secretion (1). Most cases manifest during infancy, but in 10% of patients, onset is in childhood (2). CHI is the most common cause of persistent hypoglycemia among neonates and infants, with an estimated prevalence of 1:12,000 to 50,000 live births (3, 4).

The infant's brain is vulnerable to hypoglycemia (5). If CHI is left untreated, the anabolic effect of insulin induces systemic glucose uptake in the cells and inhibits synthesis of ketone bodies and free fatty acids, resulting in brain starvation that may cause neuronal injury (6, 7). Thus, early diagnosis and proper treatment of CHI is necessary to prevent neurocognitive impairment and even death (6, 8-10). Acute treatment with glucose or glucagon aims to stabilize glucose levels during hypoglycemic episodes. Long-term therapy, which can be dietary, medical, and/or surgical, intends to prevent chronic hypoglycemia (11).

CHI is caused by disease-causing variants in genes expressed or silenced in the pancreatic beta-cell and involved in regulating insulin secretion (11). Approximately half of the cases are “channelopathies” (3, 12) caused by variants in genes encoding the beta-cell adenosine triphosphate-sensitive potassium (K_ATP_) channel (*ABCC8*, *KCNJ11*) or other channel or carrier proteins (*CACNA1D*, *KCNQ1*, *SLC16A1*) (13, 14). Patients with channelopathies typically respond poorly to diazoxide (the first-line drug) and are often challenging to treat (11).

Another CHI subtype is “metabolopathies,” in which insulin secretion is upregulated due to malfunctioning enzymes that control nutrient sensing and flux in the beta-cell (15). At least 7 genes belong to this group (*GCK*, *GLUD1*, *HADH*, *HK1*, *PGM1*, *PMM2*, *UCP2*) (11, 13). The third subtype involves variants in the genes *HNF1A*, *HNF4A, EIF2S3*, and *FOXA2* (11, 16) and may be referred to as “transcriptionopathies” (17), as these genes encode transcription factors central in defining beta-cell identity. Although molecular diagnostics is well established for CHI, a genetic etiology remains to be found in at least 20% to 30% of the patients (12, 18).

CHI is morphologically classified as diffuse or focal. Diffuse CHI is defined as a defect involving all or most pancreatic beta-cells. It is the most common form and seen in all genetic subtypes of CHI (19, 20). The focal form appears as islet adenomatosis in a limited area of the pancreas (21). It is associated with recessive, paternally inherited monoallelic *ABCC8* or *KCNJ11* variants in combination with a somatic second hit with the loss of the maternal allele in a limited pancreatic region (22, 23). For this form, surgical resection of the affected focus is usually curative (23).

Several CHI genes are also well-known causes of maturity-onset diabetes of the young and neonatal diabetes (24-26). Knowledge about the mutational panorama of beta-cell genes in a population will enable efficient diagnostics of both monogenic diabetes and CHI. In 2009, we described the distribution of *ABCC8* variants in 33 Norwegian CHI probands (27). We have now evaluated all CHI probands referred to our hospital for 2 decades and present the genetic and clinical characterization of this nationwide, population-based material.

## Methods

### Study Design and Patients

We performed a retrospective cohort study, reviewing clinical records and genetic work-up for all probands (n = 98) referred to the National Treatment Service of Congenital Hyperinsulinism at the Children and Youth Clinic, Haukeland University Hospital (HUS), Bergen, Norway. Between 2002 and June 2023, patients in Norway with persistent hypoglycemia not explained by prematurity, diabetes in pregnancy, or prenatal stress, and therefore suspected of CHI, were recommended a referral to HUS for diagnostics and treatment. All probands referred to HUS were recruited and included in the study. When available in the referral documents, information about investigations performed by other hospitals prior to recruitment was included in our evaluation.

### Ethical Approval

Patients above 16 years of age and parents of underaged patients gave written consent to participate in the study. The study was performed in accordance with the Helsinki Declaration and approved by the Regional Committee for Medical Research Ethics (REK Nord #536965).

#### Analysis of Clinical Data

In addition to descriptive variables for each proband, information was obtained about pregnancy and birth complications, clinical investigations, clinical chemistry, ^18^F-Fluorodopa positron emission tomography (^18^F-DOPA PET) scan results, initial and current treatment, final diagnosis, and status at the most recent follow-up (Table 1). Birth weights were standardized using birth weights from the Norwegian Mother, Father and Child Cohort Study (MoBa) (28) as reference. Sex and gestational age were used as covariates. For more information on the Mother, Father and Child Cohort Study cohort and standardization procedure, see Supplementary Methods (29). Macrosomia was defined as a birth weight above the 90th percentile.

**Table 1.** Clinical characteristics of 69 CHI probands

Description	Probands with a genetic finding					Clinically verified
	Total *ABCC8*	Dominant *ABCC8*	Recessive *ABCC8*	Paternal *ABCC8* with second hit	Other genetic etiology	Unsolved etiology
n (%)	39 (51)	8 (11)	15 (20)	16 (21)	7 (9)	23 (30)
Female/male	19/20	2/6	10/5	7/9	4/3	11/12
Gestation age, w (n)	38.3 ± 2.1 (37)	38.4 ± 2.4 (7)	37.7 ± 1.7 (15)	38.9 ± 2.4 (15)	39.1 ± 1.7 (7)	37.5 ± 4.0 (16)
Birth weight, g (n)	4252 ± 883 (39)	3841 ± 932 (8)	4614 ± 773 (15)	4119 ± 878 (16)	3825 ± 671 (7)	3182 ± 961 (16)
Birth length, cm (n)	52.2 ± 3.3 (30)	50.4 ± 3.4 (6)	53.6 ± 2.8 (12)	51.8 ± 3.5 (12)	51.2 ± 1.5 (5)	49.9 ± 3.3 (7)
APGAR 0/5/10 minutes (n)	7.3/8.7/9.2 (22)	8.5/9.8/9.8 (4)	6.1/7.9/8.8 (7)	7.7/8.8/9.3 (10)	8.7/9.3/9.5 (3)	7/7.5/8.5 (4)
Median age at onset [range], d (n)	0 [0-365] (36)	0 [0-365] (6)	0 [0-50] (15)	0 [0-241] (15)	3 [0-440] (7)	104 [0-378] (17)
Glycemia at onset, mmol/L (n)	1.0 ± 0.9 (26)	1.2 ± 1.2 (5)	1.0 ± 0.5 (7)	0.9 ± 1.0 (14)	1.0 ± 0.5 (6)	2.0 ± 0.7 (14)
Carbohydrate need, mg/kg/min (n)	15.7 ± 7.6 (23)	11.2 ± 4.5 (5)	17.5 ± 8.5 (8)	16.5 ± 7.9 (10)	12.6 ± 1.9 (4)	13.0 ± 7.9 (9)
Diazoxide-responsive (n)	6 (25)	3 (3)	2 (10)	1 (12)	4 (5)	11 (16)
Octreotide-responsive (n)	16 (21)	1 (1)	6 (9)	9 (11)	0 (1)	0 (2)
Morphology	13 diffuse6 focal	2 diffuse	7 diffuse	4 diffuse 6 focal	1 diffuse	3 diffuse
Surgery SP/PR	6/5	0/0	5/0	1/5	1/0	4/0
Neurologic sequela (n)	17 (34)	4 (7)	6 (13)	7 (14)	4 (6)	7 (14)
Resolution of phenotype (n)*^a^*	8 (23)	2 (6)	3 (9)	3 (8)	2 (4)	7 (13)
Deceased probands (n)	5 (39)	1 (8)	1 (15)	3 (16)	0 (7)	1 (23)

Data from the 8 patients classified as clinically uncertain CHI are not included. One proband with a genetic variant in both *GCK* and *ABCC8* is included only under “Other genetic etiology.” The diazoxide- and octreotide-responsive patients are those with satisfactory clinical effect.

Abbreviations: CHI, congenital hyperinsulinism; d, days; n, number of probands with available data; PR, partial resection; SP, subtotal pancreatectomy; w, weeks.

^*a*^Nonsurgically treated probands.

The primary diagnostic criterion was persistent hypoglycemic episodes concurrent with detectable serum insulin or C-peptide. Additional criteria used to substantiate the diagnosis were suppressed serum ketone bodies and free fatty acids, positive glucagon response test, and elevated carbohydrate need (≥ 8 mg/kg/minute) (4). Since our national treatment service is focused on CHI as a monogenic beta-cell disease and only a fraction of Norwegian patients with syndromic CHI are referred, we excluded patients with syndromic CHI from our final cohort (eg, Beckwith–Wiedemann syndrome, Sotos syndrome, Kabuki syndrome). Hypoglycemia was defined as recommended by the Norwegian Society of Pediatricians’ neonatal guidelines: blood glucose levels ≤ 1.7 mmol/L within the first 24 hours of life, ≤ 2.5 mmol/L between 24 and 48 hours of life, and ≤ 3.0 mmol/L after 48 hours of life (30). Diazoxide response was defined as resolution of persistent hypoglycemia (≤ 3 mmol/L) within 3 days of a 15 mg/kg/day dose. Patients with incomplete data were assigned a diagnosis by P.R.N. based on clinical judgment.

### Genetic Workup

Genetic testing was performed for all probands. Probands admitted before 2019 (n = 75) were initially analyzed by Sanger DNA sequencing of *ABCC8* and *KCNJ11*. If negative, other CHI-related genes were screened as indicated by the patients’ phenotype (*GCK*, *GLUD1*, *HADH*, *HK1*, *HNF1A*, *HNF4A*, *SLC16A1*). Probands referred from 2019 (n = 23) were tested using whole-exome sequencing and subsequent variant analysis based on 2 expert-curated gene panels (31, 32) that included 15 CHI-related genes (*ABCC8*, *AKT2*, *FOXA2*, *GCK*, *GLUD1*, *GPC3*, *HADH*, *HNF1A*, *HNF4A*, *INSR*, *KCNJ11*, *KDM6A*, *KMT2D*, *PMM2*, *SLC16A1*) and 15 other genes related to either syndromic hyperinsulinism, persistent hypoglycemia, or insulin resistance (*AGPAT2*, *ALMS1*, *BLM*, *BSCL2*, *CAVIN1*, *LIPE*, *LMNA*, *PCNT*, *PCYT1A*, *PIK3R1*, *PLIN1*, *POLD1*, *PPARG*, *WRN*, *ZMPSTE24*). If a pathogenic or suspected pathogenic variant was detected, parental samples were tested to determine the inheritance pattern of the variant. Siblings were tested on indication.

### *HK1* Intronic Variants

Probands with unsolved genetic etiology after routine diagnostics underwent analysis for noncoding variants in intron 2 of *HK1* (33). The conserved 42-bp region of *HK1* intron 2 (GRCh37 Chr10:71,108,645-71,108,686) was amplified by primers 5′-AGCCTGGGCAACAGAAAC-3′ (forward) and 5′-GCTACAAGCTCAGCCTCTTTC-3′ (reverse). The PCR product (397 bp) was then Sanger-sequenced with the same reverse primer and with forward primer 5′-CATTGAGCTGGACTCAGGC-3′.

Probands negative for this assay then underwent screening for the deletions within *HK1* intron 2 described in (33). Two digital droplet PCR (ddPCR) assays were developed for the Bio-Rad QX200 ddPCR System [Supplementary Table S1 (29)]. ddPCR was performed according to the manufacturer's recommendations and copy number status determined using Bio-Rad QuantaSoft software. A positive control sample was provided by Wakeling et al (33).

### Variant Interpretation

Variants were classified according to the joint consensus guidelines of the American College of Medical Genetics and Genomics and the Association for Molecular Pathology. Each variant was assigned 1 of 5 classes: benign, likely benign, variant of uncertain significance (VUS), likely pathogenic, or pathogenic (34). VUS assessed by J.M. and S.B. as having a higher likelihood of being disease-causing than benign were classified as VUS + .

### Statistical Analysis

Statistical analyses were performed using Excel version 16.72 and IBM SPSS 28. Chi-squared tests were conducted to explore associations between categoric data. The significance limit was set to *P* ≤ .05. Mean ± SD was utilized to express continuous data unless indicated otherwise. The 95% confidence interval of the birth prevalence was calculated using the incidence rate calculator in reference (35).

## Results

### Differential Diagnoses of CHI

**Figure 1.**
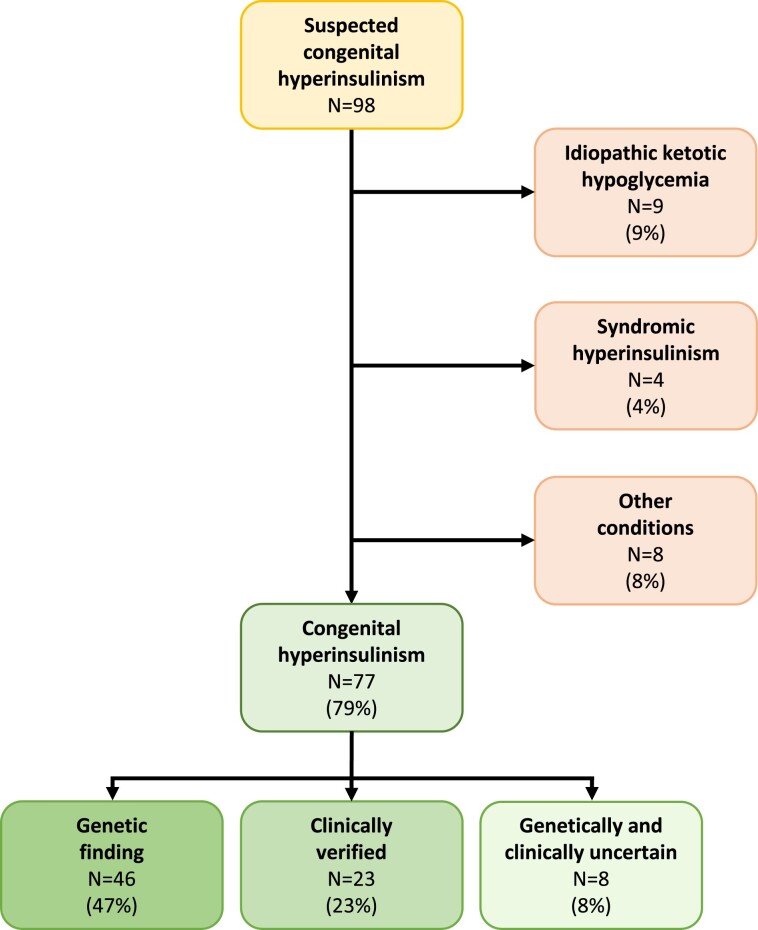
Flowchart of the study population consisting of 98 suspected CHI probands referred to Haukeland University Hospital between 2002 and 2023. Probands with idiopathic ketotic hypoglycemia, syndromic hyperinsulinism and other conditions were excluded from the study. “Genetic finding” includes probands with a mutation that justified the CHI diagnosis. “Clinically verified” probands had a phenotype that fulfilled diagnostic criteria for CHI but no pathogenic gene variant was identified. Patients with a clinical picture consistent with CHI but with insufficient clinical data to confirm the diagnosis and without genetic findings were classified as “Genetically and clinically uncertain.” Abbreviation: CHI, congenital hyperinsulinism.

We studied 98 referred CHI probands, of whom 21 were evaluated to have another diagnosis than CHI [Fig. 1; details given in Supplementary Table S2 (29)]. CHI was therefore the most common diagnosis in our cohort of neonates and children with persistent hypoglycemia, and the final cohort consisted of 77 probands.

### Profile of the CHI Cohort

There were genetic findings in 46 probands, corresponding to 47% of the whole cohort before exclusion (n = 98) and 60% of the 77 individuals with CHI (Fig. 1). Pathogenic, likely pathogenic, or VUS variants were found in 7 different genes (*ABCC8*, *GCK*, *GLUD1*, *HADH*, *HK1*, *HNF4A*, *KCNJ11*) (Tables 2 and 3). The frequency of genetic findings was similar among the probands who were analyzed by Sanger DNA sequencing (59%) and those who were screened by panel-based next-generation sequencing diagnostics (61%). Twenty-three probands had clinically verified CHI but no genetic findings. For the remaining 8 probands, CHI was suspected due to the clinical development and/or treatment response, but there was insufficient clinical data to reach a final diagnosis, and genetic testing was negative. They were classified as “genetically and clinically uncertain” (Fig. 1).

**Table 2.** Overview of pathogenic or potentially pathogenic variants of the *ABCC8* gene identified among Norwegian CHI probands

Location	Nucleotide change	Amino acid change	Mutation type	No. of probands	Inheritance	Variant interpretation	Ref.
Exon 1	c.12del	p.(Phe5SerfsTer73)	Frameshift	1	Recessive	P	(36)
Exon 1	c.62T > A	p.(Val21Asp)	Missense	8	Recessive	P	(27)
Exon 1	c.146T > C	p.(Ile49Thr)	Missense	1	Suspected recessive	LP	Novel
Exon 2	c.220C > T	p.(Arg74Trp)	Missense	1	Recessive	P	(37)
Exon 4	c.560T > A	p.(Val187Asp)	Missense	1	Recessive	P	(27)
Exon 5	c.689A > G	p.(Tyr230Cys)	Missense	1	Recessive	LP	(38)
Exon 5	c.691T > C	p.(Trp231Arg)	Missense	1	Recessive	LP	(27)
Exon 5	c.742C > T	p.(Arg248Ter)	Nonsense	1	Recessive	P	(27)
Exon 5	c.801C > A	p.(Cys267Ter)	Nonsense	1	Recessive	P	(27)
Intron 6	c.1012-3C > G	p.?	Splicing	1	Recessive	LP	(27)
Exon 7	c.1096C > T	p.(Leu366Phe)	Missense	1	Suspected dominant	LP	(38)
Exon 7	c.1123G > A	p.(Ala375Thr)	Missense	1	Suspected dominant	VUS	Novel
Exon 10	c.1468G > T	p.(Glu490Ter)	Nonsense	1	Recessive	P	(27)
Exon 10	c.1508T > C	p.(Leu503Pro)	Missense	1	Recessive	P	(27)
Exon 10	c.1513G > T*	p.(Gly505Cys)	Missense	1	Suspected recessive	VUS	(38)
Intron 10	c. 1630 + 1G > T	p.?	Splicing	8	Recessive	P	(27)
Exon 12	c.1771T > C	p.(Phe591Leu)	Missense	1	Recessive	P	(39)
Intron 15	c.2117-1G > A	p.?	Splicing	2	Recessive	P	(40)
Exon 23	c.2749C > T	p.(Gln917Ter)	Nonsense	1	Recessive	P	(27)
Exon 26	c.3259T > C	p.(Trp1087Arg)	Missense	1	Suspected dominant	VUS	Novel
Exon 28	c.3454G > A	p.(Ala1152Thr)	Missense	1	Suspected dominant	P	(41)
Exon 33	c.4034A > C	p.(Gln1345Pro)	Missense	1	Dominant	LP	Novel
Exon 33	c.4089C > A	p.(His1363Gln)	Missense	1	Suspected dominant	VUS+	(42)
Exon 34	c.4198G > A	p.(Gly1400Arg)	Missense	1	Recessive	P	(27)
Exon 35	c.4238C > T	p.(Pro1413Leu)	Missense	1	Recessive	P	(27)
Exon 35	c.4253G > A	p.(Arg1418His)	Missense	1	Recessive	P	(43)
Exon 37	c.4432G > A	p.(Gly1478Arg)	Missense	1	Dominant	P	(27)
Exon 37	c.4477C > T	p.(Arg1493Trp)	Missense	4	Recessive	P	(27)
Exon 37	c.4478G > C	p.(Arg1493Pro)	Missense	1	Recessive	LP	Novel
Exon 37	c.4532T > G	p.(Ile1511Ser)	Missense	1	Dominant	P	(44)
Exon 38	c.4591A > G	p.(Thr1531Ala)	Missense	1	Recessive	P	(27)

The exon 10 variant identified in a proband with a coexisting pathogenic *GCK* variant is indicated by an asterisk. Nucleotide reference sequence is NM_000352.6 (www.ncbi.nlm.nih.gov).

Abbreviations: CHI, congenital hyperinsulinism; LP, likely pathogenic; P, pathogenic; VUS, variant of uncertain significance; VUS+, variant of uncertain significance assessed as more likely to be disease-causing than benign.

**Table 3.** Variants identified in CHI genes other than *ABCC8* among Norwegian CHI probands

Gene	Location	Nucleotide change	Amino acid change	Mutation type	No. of probands	Inheritance	Variant interpretation	Ref.
*GCK*	Exon 10	c.1367C > T	p.(Ala456Val)	Missense	1	Dominant	P	(45)
*GLUD1*	Exon 7	c.953G > C	p.(Arg318Thr)	Missense	1	Dominant	LP	Novel
	Exon 11	c.1493C > T	p.(Ser498Leu)	Missense	1	Dominant	P	(46)
*HADH*	Exon 5	c. 547-3_549del	p.?	Exon skipping due to deletion	1	Recessive	P	(47)
*HK1*	Intron 2	c.226 + 4909A > C	–	Noncoding	1	Dominant	VUS+	Novel
*HNF4A*	Exon 4	c.421C > T	p.(Arg141Ter)	Nonsense	1	Dominant	P	(48)
*KCNJ11*	Exon 1	c.866G > A	p.(Gly289Asp)	Missense	1	Dominant	LP	Novel

Nucleotide reference sequences are *GCK* (NM_000162.5), *GLUD1* (NM_005271.5), *HADH* (NM_005327.7), *HK1* (NM_000188.3), *HNF4A* (NM_175914.5), *KCNJ11* (NM_000525.4) (www.ncbi.nlm.nih.gov).

Abbreviations: CHI, congenital hyperinsulinism; LP, likely pathogenic; P, pathogenic; VUS, variant of uncertain significance; VUS+, variant of uncertain significance assessed as more likely to be disease-causing than benign.

Table 1 presents a summary of clinical data of the 69 probands with verified CHI. Gestation age was known in 60 probands. Most probands (80%) were born to term, ie, in week 37 or later. Seventeen of 40 (43%) nonsurgically treated probands of any etiology with data on follow-up had spontaneous resolution of their phenotype, with the median age at last follow-up being 7.6 years. None of the nonsurgically treated probands had developed diabetes. Six probands (8%) were deceased, with 5 having *ABCC8* variants and 1 without genetic findings. The majority of the CHI cohort were single cases, but 6 probands also had affected family members. Two consanguineous families were identified. The parents were either first or double first cousins, and their affected children carried homozygous variants in *ABCC8* and *HADH* (47), respectively. Seventeen probands had at least 1 parent of non-Norwegian descent.

### Probands With *ABCC8* Variants

Altogether, 40 probands had a variant in *ABCC8* interpreted as pathogenic, likely pathogenic, or VUS (52% of the CHI cohort; 87% of probands with a genetic finding). Thirty-one unique variants were identified, the majority being missense (Table 2). Five variants were previously undescribed [p.(Ile49Thr), p.(Ala375Thr), p.(Trp1087Arg), p.(Gln1345Pro), p.(Arg1493Pro)]. The 3 most frequent variants were the splice variant c.1630 + 1G > T and the missense variants p.(Val21Asp) and p.(Arg1493Trp), independently observed in 8, 8, and 4 families, respectively. These recurrent variants are considered founder variants in Norway.

**Figure 2.**
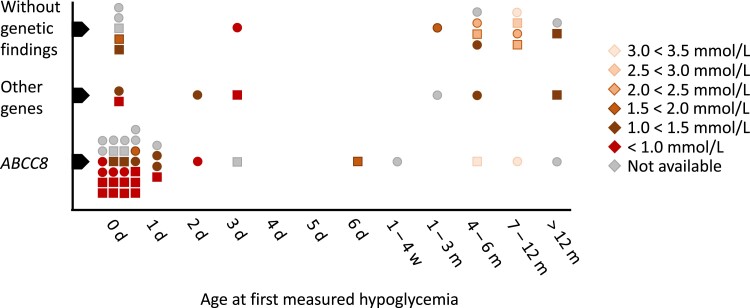
Age and glycemia at time of disease onset in 60 CHI probands. The x-axis indicates disease onset ascertained as age at first measured hypoglycemia. Glucose levels at first measured hypoglycemia are color-coded according to the legend. Squares correspond to males and circles to females. For some probands, the measured glucose value at disease onset was not available (grey color). Abbreviations: d, days; m, months; w, weeks.

*ABCC8* probands generally had a more severe disease debut with low glycemia levels and younger age at disease onset than the rest of the cohort (Fig. 2). One proband had a coexisting pathogenic *GCK* variant and is described separately later. When the time of onset was known, 32/36 (89%) had disease onset within the first week of life and 25/36 (69%) within the first day. Birth weight was available for all 39 probands (Table 1), of whom 24 (62%) were macrosomic. Data on medical treatment was available in 33 of the probands. Diazoxide treatment was initiated in 27, resulting in response in 6 (24%), partial effect in 3 (12%) and no effect in 16 (64%), whereas treatment was stopped in 2 probands due to severe side effects. In the 20 probands with data on delivery method, 7 (35%) were delivered by cesarean section because of large gestational weight or other pregnancy/birth complications.

Eight probands had dominant inheritance of their *ABCC8* variant, and in 3 cases the variant had arisen de novo. Although 4 probands had onset within the first day of life, the dominant subgroup showed a milder phenotype as only 3 patients needed drug therapy and were responsive to diazoxide. One had severe side effects and had to switch to octreotide, which was efficient. The carbohydrate need was available for 5 probands and was elevated in 3 (mean = 11.2 mg/kg/min).

Recessive homozygous (n = 6) or compound heterozygous (n = 9) *ABCC8* variants were detected in 15 probands. Fourteen of these had disease onset within the first week of life, including 11 on the first day. Seven of 8 probands with data had an elevated carbohydrate need (mean = 17.5 mg/kg/min). Data on medical treatment were available in 13 probands. Diazoxide was commenced in 11: 2 responsive, 2 partially responsive, 6 unresponsive, and 1 proband had the drug terminated due to severe side effects. In 9 probands with initiation of octreotide, 6 exhibited a response. Pasreotid and sirolimus were used in 2 probands with adequate effect. Five patients underwent a subtotal pancreatectomy, and all had developed diabetes when evaluated after 10 years of age.

Monoallelic recessive paternal inheritance with presumed somatic loss of the maternal allele in a focal or multifocal endocrine pancreatic region was, including the proband with a coexisting *GCK* variant described later, seen in 17 probands (22%), constituting the largest *ABCC8* subgroup in our cohort. Age at onset was known for all but 1 proband, and 14/15 had disease onset within the first week of life, including 10 on the first day. Only 1 patient was responsive to diazoxide. Among 11 octreotide-treated probands, 9 were responsive and 1 was partially responsive. Nine probands underwent ^18^F-DOPA PET imaging revealing focal morphology in 5. One proband had an autopsy where focal morphology was uncovered after death, resulting in 6 total cases of focal CHI (8% of the 77 CHI probands).

### Probands With Other Genetic Findings

*KCNJ11*: The novel heterozygous missense variant p.(Gly289Asp) in *KCNJ11* was detected in one proband with normal birth weight (Table 3). Hypoglycemia (blood glucose 1.2 mmol/L) and seizures occurred on the second day of life. The carbohydrate need was 12.3 mg/kg/min. Treatment involved dietary adjustments.

*GLUD1*: Heterozygous *GLUD1* gain-of-function variants are associated with hyperinsulinism-hyperammonemia syndrome (49). We observed de novo missense *GLUD1* variants in two probands (Table 3). Normal birth weights and elevated ammonia levels were reported in both probands (several measurements ranging from 100 to 300 µmol/L). Disease onset with seizures was in the 3rd and 15th month, respectively, for the probands harboring the pathogenic p.(Ser498Leu) variant and the novel, likely pathogenic p.(Arg318Thr) variant. Treatment involved dietary adjustments in combination with diazoxide. Carbohydrate need was available in 1 proband (14 mg/kg/min).

*GCK*: A de novo variant [p.(Ala456Val)] in *GCK* that coexisted with a heterozygous paternal *ABCC8* variant of uncertain significance [p.(Gly505Cys)] was discovered in 1 macrosomic proband with hypoglycemia (0.1 mmol/L) on the first day of life (Tables 2 and 3). Dietary adjustments together with diazoxide and octreotide had insufficient effect. ^18^F-DOPA PET imaging displayed diffuse morphology, leading to a subtotal pancreatectomy. Exocrine insufficiency followed the surgery.

*HADH*: A homozygous deletion in *HADH* was identified in 1 proband with normal birth weight and consanguineous parents (Table 3). This family has been published by us previously (47).

**Figure 3.**
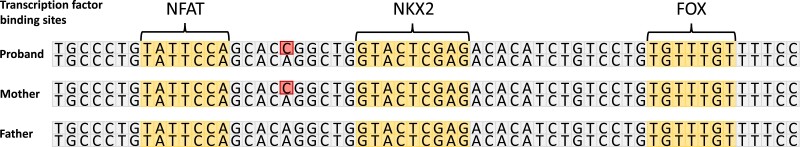
Localization of a novel noncoding *HK1* variant (c.226 + 4909A > C) found in intron 2. On top of the sequences are binding motifs of transcription factor families according to Wakeling et al (33). DNA sequence is taken from nucleotide reference sequence NM_000188.3 (www.ncbi.nlm.nih.gov).

*HK1*: One proband with normal birth weight was heterozygous for a novel, maternally inherited VUS + variant in *HK1* (Table 3). The variant (c.226 + 4909A > C) was present in a conserved 42-bp region of intron 2 (Fig. 3), close to other reported variants in this region (33). Disease onset with seizures was relatively late at age 5.5 months with plasma glucose 1.3 mmol/L. The proband was diazoxide-responsive. The clinical status of the mother was not available. The frequency of intronic *HK1* variants in our national CHI cohort is ≈1% (1/77) and ≈3% (1/36) of those without a genetic diagnosis when *HK1* screening was performed.

*HNF4A*: A heterozygous variant in *HNF4A* [p.(Arg141Ter)] was identified in 1 proband (Table 3) with macrosomia. Hypoglycemia (1.4 mmol/L) presented during the first day of life with symptoms of irritability and head lag. The proband was hypersensitive to diazoxide.

### Genetically Unsolved Probands

After genetic testing and clinical evaluation, 23 probands had clinically verified CHI but unsolved genetic etiology. Three of 16 probands (19%) with available birth weight were macrosomic. Probands with known genetic etiology were significantly more likely to have disease onset within the first week of life than those with unsolved genetics [χ^2^ (1, n = 60) = 13.6, *P* < .001] (Fig. 2). An elevated carbohydrate need was found in 6 of 9 unsolved probands (mean = 13.0 mg/kg/min). Among 17 probands with data on medical treatment, diazoxide was instigated in 16 (11 responsive, 2 partially responsive, 3 unresponsive). In a recent control of 3 probands who underwent a subtotal pancreatectomy, 2 had developed diabetes and 1 had elevated but not diabetic hemoglobin A1c values.

### Neurologic Sequela

Neurologic sequela was a recurrent characteristic of the entire CHI cohort. Neurologic status was available at hospital discharge or follow-up in 58 probands. Of these, 31 (53%) were reported by parents or a physician to have neurologic sequela (1 or more of the following: developmental delay of any character and degree, n = 17; suspected or confirmed epilepsy, n = 12; concentration or learning disabilities, n = 7; damage of brain tissue, n = 7; multifunctional disabilities, n = 2; visual, n = 3; or hearing n = 1 impairment). The age of neurologic debut was generally unknown for the probands. However, the median age at last follow-up was 10.2 years for the neurologically affected probands. Symptomatic data of hypoglycemia were available in 24 of the probands with neurologic sequela and in 26 of the neurologically unaffected probands. Seizures were 3-fold as common among patients who had developed neurologic sequela (14/24, 58%) compared to those who did not (5/26, 19%) [χ*^2^* (1, n = 50) = 8.2, *P* < .01]. In contrast, the occurrence of reduced or loss of consciousness was equally prevalent in both groups (21% vs 19%, respectively). In the 5 probands where both seizures and reduced or loss of consciousness occurred, 4 had developed neurologic sequela. We found no correlation when comparing the number of probands developing neurologic sequela in subtotally pancreatectomized (4/5) (median age at last follow-up: 14.8 years) against nonpancreatectomized (21/43) (median age at last follow-up: 7.6 years) probands.

### Birth Prevalence

Twenty-nine of the CHI probands (16 with a genetic finding, 11 clinically verified, 2 genetically/clinically uncertain) were born during the past decade (2013-2022). In this period, mothers in Norway gave birth to 562 794 children (50). Thus, we conclude that the minimum Norwegian birth prevalence of CHI is 1:19 400 live births (95% confidence interval 1:13.500; 1:27 900).

## Discussion

In this study, we have evaluated all probands with suspected CHI referred to the National Treatment Service of Congenital Hyperinsulinism in Norway over the past 2 decades. Having a national treatment service within a public health system enables investigation of a broad spectrum of disease in one country and is considered one of the study's strengths.

### Genetic Findings

In our CHI cohort, 7 different genes had variants that we consider disease-causing (*ABCC8*, *GCK*, *GLUD1*, *HADH*, *HK1*, *HNF4A*, *KCNJ11*), and a genetic diagnosis was reached in 60% of the CHI probands. We identified 5 previously undescribed variants in *ABCC8* and 1 in each of *GLUD1*, *HK1,* and *KCNJ11*. Until now, only *ABCC8* and *HADH* variants have been reported in Norwegian CHI patients (27, 47). Variants in the *ABCC8* gene are by far the most common cause of CHI in our study population, which is in line with larger patient cohorts (18, 51).

We searched specifically for noncoding variants of *HK1* intron 2 in 37 CHI probands that were not genetically solved. We identified the novel and possibly disease-causing (VUS+) variant c.226 + 4909A > C. Unfortunately, glycemic status or medical history was unknown for the mother, who also carried the variant. However, the base substitution has occurred within the conserved 42-bp region of intron 2 (33), in a nucleotide position known to be affected in unpublished CHI patients (S. Flanagan, personal communication). We detected no CHI probands carrying a deletion that encompassed the conserved intronic *HK1* region (33). Our results suggest a frequency of disease-causing variants in intron 2 of the *HK1* gene to be in the order of 1%.

We also discovered 1 CHI proband with potentially 2 mutated genes. This severely affected individual harbored a de novo heterozygous activating variant in *GCK* [p.(Ala456Val)] as well as a heterozygous, paternally inherited *ABCC8* variant [p.(Gly505Cys)] considered a VUS. The *GCK* variant was first identified by Christesen et al in a diazoxide-responsive patient with onset in the neonatal period (45). Contrary to that patient, our proband had onset within the first day of life with unmanageable hypoglycemia. He responded neither to diazoxide nor octreotide, and ^18^F-DOPA PET imaging revealed diffuse morphology. This led to a subtotal pancreatectomy. Whereas we consider the *GCK* variant as the underlying cause for disease in this proband, we speculate that the *ABCC8* variant contributes to the severe phenotype with unresponsiveness to medical treatment.

### Clinical Aspects

Loss-of-function of *ABCC8* was associated with the most severe phenotype in our cohort. Probands with pathogenic or likely pathogenic *ABCC8* variants generally had very early disease onset, with 89% having manifested within the first week of life (Fig. 2) and only 24% being responsive to diazoxide. Our data support the literature as *ABCC8* variants tend to be unresponsive to medical treatment and to have early and severe disease onset (52, 53). All 8 probands who had undergone subtotal pancreatectomy and for whom follow-up data were available had developed diabetes, except 1 case who had elevated but not diabetic hemoglobin A1c values. The high frequency of diabetes is consistent with Arya et al (54), who studied 45 children treated with subtotal pancreatectomy. We observed resolution of the phenotype in 43% of nonsurgically treated CHI probands of any etiology (Table 1). None had developed diabetes, possibly explained by young age at follow-up. These findings are supported by a recent study of 18 nonpancreatectomized, recessive *ABCC8* probands of whom 70.6% experienced spontaneous resolution. Thus, medical therapy should always be considered as a long-term treatment alternative instead of subtotal pancreatectomy (55). However, in accordance with international CHI guidelines, surgery may be required in the most severe cases (56).

Huopio et al were the first to describe a dominant *ABCC8* family (57). Affected members had a mild phenotype that could be managed on diazoxide. Similarly, Pinney et al showed that 15 of 16 probands with dominantly inherited K_ATP_-channel defects were well controlled by diazoxide (58). Likewise, we found dominant inheritance of pathogenic *ABCC8* variants to result in a milder phenotype than recessive inheritance. In probands with dominantly inherited variants, only 50% required drug treatment, all of whom responded to diazoxide.

Verified focal morphology comprised only 8% of the CHI probands. Similar frequencies were observed in Kapoor et al (5.3%) (51) and Männistö et al (8.5%) (12) when studying 300 and 153 CHI patients, respectively. Considering that disease morphology remained unknown in 7 of the 17 recessive monoallelic paternally inherited *ABCC8* probands in our cohort, it is likely that 8% is lower than the real prevalence of focal morphology. Also, the absence of a focal lesion on PET does not exclude the possibility of identifying a focal lesion during surgery if the lesion is < 5 mm or adjacent to the kidney (59). On the other hand, reports with considerably higher frequency, such as Snider et al (18), who found 35.7% of 417 patients, could represent an overestimation caused by selection bias. International treatment centers may receive patients from the more severe side of the clinical spectrum. Since K_ATP_-channel defects are the cause of both focal CHI and the phenotypically most severe cases of CHI, they might be overrepresented in such centers. Notably, focal morphology was investigated by ^18^F-DOPA PET (or by autopsy) in 23 of our probands with various etiologies but remained exclusively associated with monoallelic, paternally inherited *ABCC8* probands.

Probands with unsolved genetic etiology had a later disease onset than probands with verified genetic etiology. Hewat et al reported that elevated birth weight, diazoxide unresponsiveness, and onset within the first week of life were independently associated with K_ATP_-channel defects and that diazoxide unresponsiveness combined with elevated birth weight could predict these variants (53). In support, we found that the probability of identifying a genetic etiology decreases if disease onset is after 1 week of life (Fig. 2).

Of the CHI probands with follow-up data, the majority (53%) developed neurologic sequela of any character and degree, aligning with previous studies (6, 8, 60). Additionally, Banerjee et al list in their review neurocognitive impairment in 24% to 48% of CHI individuals (61). The long follow-up of our cohort may contribute to the high prevalence of neurologic affection. Nonetheless, the low age of the neurologically affected probands (median age 10.2 years) suggests that onset of neurologic sequela occurs early in a CHI patient’s lifespan. Interestingly, our investigation revealed that probands who had experienced seizures were 3-fold as likely to develop neurologic sequela compared to those without seizures. Notably, 4 of the 5 probands who had experienced both seizures and loss of/reduced consciousness eventually developed neurologic sequela. Because we have not evaluated severity or length of the seizures, we speculate that those who had seizures without developing neurologic sequela had fewer, shorter, and milder seizures. Clinically, we stress the importance of avoiding seizures and unconscious episodes in patients with CHI. Newly diagnosed individuals should achieve stable and near-normal metabolic control with mean glucose over 3.3 mmol/L at discharge. Today it is possible to better monitor this by taking advantage of continuous glycemic measurement devices (62). We found no significant correlation between development of neurologic sequela in probands who underwent a subtotal pancreatectomy vs nonpancreatectomized probands. We ascribe this to these pancreatectomies being initiated after severe hypoglycemic episodes (and possibly seizures) have occurred, too late to prevent neurologic affection.

### Birth Prevalence

In 2009, we estimated the minimum birth prevalence of CHI in Norway to be 1:70 000 based on *ABCC8* variants only (27). Due to having a single national treatment center, the present cohort is likely to represent the large majority of CHI occurring in Norway. During the last 10 years, national awareness about CHI has increased, and more systematic referral procedures have been implemented. Hence, we used data only from the last decade to estimate the birth prevalence of the disease, which is 1:19 400 live births. In comparison, other studies have reported a birth prevalence ranging from 1:12 000 and up to 1:50 000 (3, 4, 11, 13). However, 1:19 400 must be the *minimum* birth prevalence of CHI in Norway as we cannot exclude the possibility of unrevealed or misdiagnosed probands. Moreover, if our estimate also had included affected relatives of the CHI probands and cases of syndromic CHI, the prevalence would have been higher. There might also be individuals born in the past decade who will not develop a CHI phenotype until childhood, although this is likely to apply to only a few individuals (2).

### Differential Diagnoses

Idiopathic ketotic hypoglycemia occurred in 9% of the probands and was the most common differential diagnosis, followed by syndromic hyperinsulinism (4%). Our observation aligns with the review of Drachmann et al (63) who found idiopathic ketotic hypoglycemia to be the most common cause of transient neonatal hypoglycemia. We underline the clinical importance of establishing early if there are normal or elevated levels of ketone bodies concurrent with hypoglycemia.

### Limitations

Our study has several limitations. Not all probands were tested by a gene panel, and only *ABCC8* and *KCNJ11* were sequenced for all probands. Thus, we may have missed some genetic diagnoses and possibly underestimated the prevalence of nonchannel CHI genes in the cohort. Gene-panel testing also has its limitations and will not detect variants in noncoding DNA or large deletions/insertions. In addition, we did not perform functional studies of the novel variants to determine the pathogenicity. For some probands, both parents were not available for segregation analysis, and the genetic inheritance pattern could not be determined. Moreover, our study is retrospective. Some of the records were incomplete, and for some probands little clinical data were available. Finally, we did not systematically perform neurologic or neuropsychological examination. Hence, the nuances and prevalence of these features can be underestimated in our cohort.

## Conclusions

Congenital hyperinsulinism in Norway is caused by defects in 7 different genes and has a minimum birth prevalence of 1:19,400. *ABCC8* is by far the most common cause and generally results in the most severe phenotypes with regard to age of onset, metabolic control, and treatment response. In the most severe cases with diffuse morphology, conservative medical treatment could be a viable option in place of a subtotal pancreatectomy. If onset is after the first week of life, it is less likely that a genetic etiology will be identified. Finally, we propose a possible correlation between seizures during hypoglycemia and subsequent development of neurologic sequela.

## Data Availability

Original data generated and analyzed during this study are included in this published article or in the data repositories listed in References.
